# Non-muscle invasive bladder cancer biomarkers beyond morphology

**DOI:** 10.3389/fonc.2022.947446

**Published:** 2022-08-03

**Authors:** Camilla De Carlo, Marina Valeri, Devin Nicole Corbitt, Miriam Cieri, Piergiuseppe Colombo

**Affiliations:** ^1^ Department of Pathology, IRCCS Humanitas Research Hospital, Milan, Italy; ^2^ Department of Biomedical Sciences, Humanitas University, Milan, Italy

**Keywords:** prognostic, predictive, biomarker, non-muscle invasive bladder cancer, bladder cancer, molecular subtypes, BCG response

## Abstract

Non-muscle invasive bladder cancer (NMIBC) still represents a challenge in decision-making and clinical management since prognostic and predictive biomarkers of response to treatment are still under investigation. In addition to the risk factors defined by EORTC guidelines, histological features have also been considered key variables able to impact on recurrence and progression in bladder cancer. Conversely, the role of genomic rearrangements or expression of specific proteins at tissue level need further assessment in NMIBC. As with muscle-invasive cancer, NMIBC is a heterogeneous disease, characterized by genomic instability, varying rates of mutation and a wide range of protein tissue expression. In this Review, we summarized the recent evidence on prognostic and predictive tissue biomarkers in NMIBC, beyond morphological parameters, outlining how they could affect tumor biology and consequently its behavior during clinical care. Our aim was to facilitate clinical evaluation of promising biomarkers that may be employed to better stratify patients. We described the most common molecular events and immunohistochemical protein expressions linked to recurrence and progression. Moreover, we discussed the link between available treatments and molecular drivers that could be predictive of clinical response. In conclusion, we foster further investigations with particular focus on immunohistochemical evaluation of tissue biomarkers, a promising and cost-effective tool for daily practice.

## 1 Introduction

Bladder cancer (BC) is diagnosed in approximately 85,000 people every year in the United States (1). Of these, nearly three quarters will predictably harbor a non-muscle-invasive bladder cancer (pT1, NMIBC), a category representing a very heterogeneous population (2). In fact, while these individuals have an elevated probability to recur or progress to muscle-invasive or metastatic disease, their outcomes may be highly variable and unpredictable (3). In general, more than half of BC will recur within 1 year after the first surgery, and will progress to a muscle-invasive bladder cancer (MIBC) in 10-30% of cases (4). Patients with NMIBC are monitored with cystoscopies over many years, inducing heavy costs for healthcare systems and discomfort for patients. In this setting, biological markers for stratifying patient risk, management, and treatment are needed. The heterogeneous morphological spectrum of urothelial carcinoma includes papillary and flat lesions, divergent differentiation (squamous and glandular) and histological variants such as nested, plasmacytoid, micropapillary, sarcomatoid and small-cell (neuroendocrine) carcinoma (5). At the molecular level, as with muscle-invasive cancer, NMIBC is also a heterogeneous disease, characterized by genomic instability, varying rates of mutation, and a wide range of protein tissue expression. In this context, genomic profiling has been shown to facilitate bladder cancer classification into molecular subtypes for a more precise patient stratification according to prognostic and predictive variables, and to advance our understanding of bladder cancer biology. More recently, molecular intertumoral heterogeneity has been investigated, leading to the identification of distinct molecular classes beyond histopathological classification and with differences in clinical outcomes. Among NMIBC, some authors have focused on genes involved in cell cycle regulation, chromatin remodeling and receptor tyrosine kinase signaling (6).

In this review, we focused on recent and past English-language literature discussing predictive and prognostic tissue biomarkers beyond morphology, which could be associated with disease recurrence and progression. We aim to facilitate clinical evaluation of promising biomarkers that may be employed to better stratify patients with NMIBC disease.

## 2 Selection criteria

For prognostic variables, a review of the published literature was performed using the Mesh terms and key words deriving from the concepts “biomarker,” “prognostic,” “tissue,” and “non-muscle invasive bladder cancer (NMIBC)”, in the period 2000-2022. For predictive variables, a review of the published literature was performed using the Mesh terms and key words deriving from the concepts “biomarker,” “predictive,” “tissue,” and “non-muscle invasive bladder cancer (NMIBC)”, in the period 2015-2022. The search was filtered for articles in English. Overall, our search strategy retrieved 203 articles (58 predictive and 145 prognostic articles). Articles from review, abstract, case reports, or discussing upper tract urothelial cancer, muscle invasive bladder cancer (MIBC), were excluded. After a first selection, the full text of 123 original articles was analyzed and finally 96 articles were included in this review.

## 3 Prognostic biomarkers in NMIBC

### 3.1 P53 and other cell-cycle biomarkers

The cell-cycle leads to cell-division and DNA replication; it is a complex and highly controlled process in which promoters and inhibitors interact to prevent uncontrolled proliferation. Mutations in cell-cycle modulators such as *p53*, *p16*, *p21* and *p27* are the most imputed in the cancerous process. *P53* is considered a crucial tumor suppressor gene, encoding for a protein (p53) that plays a central role in arresting the cell cycle, activating DNA repair, and inducing apoptosis. Immunohistochemistry represents a surrogate to the analysis of *p53* mutational status since nuclear immunoreactivity is highly concordant with genomic mutations (7–9). Indeed, proteins encoded by mutated *p53* have prolonged half-life with an increased nuclear accumulation, which is responsible for immunohistochemical detection (10).

The prognostic role of the expression of p53 in NMIBC has been largely investigated during the last 30 years, but the first publications reported controversial results (11–14), possibly due to the lack of consensus on staining protocols and on interpretation of p53 positivity. Moreover, tumor heterogeneity and complexity of downstream pathways contributing to the cancerous process raised the need to evaluate panels of multiple cell-cycle biomarkers (15, 16),. In 2007, Shariat et al. investigated the abnormal expression of p53, p21, p27 and pRB on 74 TURB NMIBC specimens. They demonstrated that all biomarkers were independently associated with progression and their combination had a cooperative effect in stratifying patients into different risk groups for tumor recurrence and progression. Moreover, the risk increased incrementally with the number of altered biomarkers (17). Later, the assessment of p53, p27 and Ki67 showed a significant predictive value for recurrence free survival (RFS) and cancer specific survival (CSS) in patients with pT1 disease at radical cystectomy (18).

However, the very first prospective trial investigating the role of cell-cycle modulators and proliferation index in NMIBC failed to find any association of p53 with progression free survival (PFS), RFS, or CSS in a series of 207 patients. The only independent predictors were MIB1 score and tumor grade (19).

Shariat et al. found no independent association of cyclin D1 and cyclin E1 with recurrence or progression in a multivariate analysis of NMIBC. Interestingly, a low expression of cyclin E1 was significantly related to altered expression of other biomarkers in the panel (p53, pRb, Ki67 and survivin) (20). In contrast, Lopez-Beltran et al. described an independent predictive value of Cyclin D1 at multivariate analysis for PFS (RR 1.009, 95%CI 1.004-1.014, *p*<0.001) and Cyclin D3 for PFS (RR 1.005, 95%CI 1.002-1.008, *p*=0.001), RFS (RR 1.003, 95%CI 1.000-1.005, *p*=0.02) and OS (RR 1.013, 95%CI 1.004-1.022, *p*=0.005), in a series of 51 pT1 high grade (HG) NMIBC (21).

A more recent prospective study conducted on 87 TURB specimens of any pT stage demonstrated that a panel of biomarkers, including p53, p21, p27, Ki67 and cyclin E1, was an independent predictor of T-stage upstaging (HR 3.3, *p*=0.02). The same was not true for T-stage combined with N-stage or N-stage alone (HR 2.76, *p*=0.06) (22).

Testing the same panel in 131 patients with high-grade NMIBC, a prospective investigation showed no correlation with RFS or PFS and no significant improvement of the risk stratification performed by EORTC (European Organization for Research and Treatment of Cancer) risk tables when tested together (23).

In 2016, Raspollini et al. successfully validated their biomarker-based risk score (galectin-3, CD44, E-cadherin, CD138, p16, Survivin, HYAL-1 and topoisomerase-IIα) with the purpose of improving the EORTC risk stratification for patients with a single T1 HG BC of less than 3 cm in diameter. The score proved a significant predictive value for those patients (24).

In a prospective series, Passoni et al. found a high concordance rate (between 77.5% and 92.2%) for the expression of p53, p21, p27, Ki67 and cyclin E1 between TURBT and matched radical cystectomy specimens. P53 and p21 showed the lowest concordance rate, while cyclin E1 showed the highest, suggesting that this correlation could help in risk stratification of patients (25).

In a series of 86 CIS, Shariat et al. demonstrated that combined p53/p21 expression status predicts RFS (HR 3.14, *p*=0.022), PFS (HR 2.94, *p*=0.042) and CSS (HR 6.89, *p*=0.031). P53 alone failed to significantly predict clinical outcome, while p21 showed an association with recurrence (HR 2.6, 95%CI 1.1-6.2) and progression (HR 4.1, 95%CI 1.1-14.7) (26).

A recent study on 134 patients with papillary NMIBC showed a significant relationship between recurrence and intensity of nuclear staining for p53 (HR 1.417, 95% CI 1.001–2.007, p = 0.049) in multivariate analysis (27).

Altogether, the aberrant expression of p53 and cell-cycle biomarkers show a negative prognostic value for patients’ outcome, especially when assessed in comprehensive panels. Nevertheless, their possible role in implementing risk stratification needs further validation.

### 3.2 Ki67

Ki67 is a nuclear protein and a marker of cell proliferation expressed in the growth fraction of neoplastic cells. Its positivity has been correlated to poor survival and recurrence in different cancer types but has not yet been confirmed in NMIBC. Ki67 has been demonstrated to predict recurrence and progression in bladder cancer, but the heterogeneous staining methods and thresholds of positivity make the direct comparison of the studies inapplicable and clinical use not reliable (28).

A study conducted on 164 pTa/pT1 BC showed that Ki67 was an independent predictor of RFS (RR 3.03 *p*=0.0005), PFS (RR 3.38, *p*=0.0162) and cancer specific survival (RR 3.81, *p*=00195) (29).

In 2009, a paper including 80 patients with pT1 NMIBC treated with radical cystectomy (RC) demonstrated that Ki67 predicted a worse RFS (HR 3.96, *p*=0.021) and CSS (HR 6.23, *p*=0.009) (17). However, this single-center retrospective study was not confirmed by a subsequent prospective multicentric external validation (22).

Ding et al. investigated the possible value of combining Ki67 staining and EORTC risk scores. Interestingly, Ki67 showed a predictive role alone and it was able to improve the EORTC scores prediction of recurrence and progression (*p*=0.0001) (30).

In more recent years, Ki67 was both confirmed and confuted as a predictor of RFS (27) (31–33).

Overall, Ki67 may be correlated to more aggressive neoplasms, with a tendency to recur. However, a reproducible employment in clinical practice to predict NMIBC behavior is difficult to obtain due to the heterogeneity of cut-offs used in the literature.

### 3.3 Cell signaling

#### 3.3.1 Fibroblast growth factor 3

Fibroblast growth factor receptor 3 (FGFR3) is a tyrosine kinase receptor that plays a role in different processes, such as cellular differentiation, growth and angiogenesis. Interestingly, a mutually exclusive distribution of *FGFR3* and *p53* mutations has been observed in urothelial carcinoma, possibly characterizing two distinct and alternative genetic pathways in its carcinogenesis (34).

In 2005, a retrospective study including 119 pT1 high grade BC – 16.8% with *FGFR3* alterations and 65.5% with *p53* mutations – did not detect any association of the mutated genes with RFS, CSS or clinicopathologic features (35). Later, the same authors conducted a large prospective study (772 patients) showing that *FGFR3* mutations were more frequently detected in low-risk BC (*p <*0.001). However, at stratified analysis, *FGFR3* status was independently associated with recurrence in TaG1 BC (HR 2.12, *p*= 0.004); no association was found with PFS and OS (36). In contrast, a recent study linked high expression of FGFR3 mRNA with a shorter RFS but a better OS (37).

Overall, tumors with loss of FGFR3 protein seem to have a more favorable presentation, with a lower stage and grade at diagnosis, when compared to both tumors without FGFR3 protein loss and neoplasms with p53 overexpression. However, it has been demonstrated that FGFR3 protein expression is more frequent than the activating mutation, especially in pT1 stage. Hence, immunohistochemistry does not reflect the *FGFR3* mutation load (38). Moreover, BC shows a heterogeneous intratumoral expression of *FGFR3* mutations (39). Altogether, these factors may be responsible for discordant results.

#### 3.3.2 HER2

The activation of human epidermal growth factor receptor 2 (HER2) triggers different intracellular pathways promoting cell proliferation, survival and mobility. Its role as a negative prognostic factor and therapeutic target has been extensively investigated in breast and gastric cancer (40–42). As for NMIBC, several studies assessing the prognostic role of HER2 expression provided conflicting results. Early studies failed to find a significant association with outcome, including recurrence and progression (43, 44),, although patients with HER2 status 3+ were found to have stage >pT2 and nodal involvement at cystectomy after progression to MIBC (45). In contrast, Chen et al. demonstrated in a study conducted on 400 NMIBC that *HER2* amplification evaluated on tissue microarray (TMA) by immunohistochemistry (IHC) and fluorescence *in situ* hybridization (FISH) was an independent predictor of recurrence and progression, and significantly associated with tumor grade (46). Similarly, *HER2* amplification assessed by silver-enhanced *in situ* hybridization (SISH) and IHC proved the same prognostic role for PFS (47, 48),. HER2 mRNA levels by real-time quantitative PCR were investigated in a large cohort of low and high grade pT1 bladder carcinomas with and without associated CIS. High HER2 expression was able to stratify patients with concomitant CIS into a lower-risk and a higher-risk group with 90% of 5-years PFS and 55% of 5-years PFS, respectively (49). The same group found a significant association between high levels of HER2 expression and PFS (50). In 2017, Cormio et al. compared HER2 IHC overexpression in BCG-treated and non-BCG-treated pT1 high-grade NMIBC. HER2 overexpression was an independent predictor of RFS and PFS in both univariate (RFS *p*=0.0013; PFS *p*=0.0322) and multivariate (RFS *p*=0.001; PFS *p*=0.041) analysis (51). In a further study, the same group demonstrated that HER2 overexpression predicted outcome in combination with microsatellite instability markers (MLH1 and MSH2) (52). More recently, Sikic et al. assessed 80 pT1 NMIBC and demonstrated that female patients with high HER2 expression had worse CSS (*p*= 0.011) and OS (*p* = 0.042), while RFS was significantly shorter in both sexes (37).

In conclusion, *HER2* status might be helpful in identifying patients with NMIBC who may benefit from a closer surveillance. Nevertheless, evidence is still controversial due to several factors, including tumor heterogeneity, varying assessing methods and different employed techniques.

### 3.4 Cadherins

Cadherins mediate cell to cell adhesion and are involved in the formation of cell junctions. Within the cadherins family, E-cadherin expression is associated with a non-invasive phenotype of tumor cells. Indeed, suppression of E-cadherin expression represents a hallmark of epithelial-to-mesenchymal transition (EMT), the major process responsible for cancer cell dissemination and metastasis (53). The switch in expression to other cadherins has been linked to a more aggressive behavior in BC (54). The first members of this family to be investigated were E-, N- and P-cadherin (55). In urothelial cancer, P-cadherin is heterogeneously expressed, showing only basal positivity in G1-G2 tumors and a pantumor positivity in G3 (56).

In NMIBC, several groups have investigated the role of different subclasses of cadherins. Wang et al., in a cohort of 110 patients, found an association between high expression of P-cadherin and a worse PFS (*p=* 0.034); moreover, P-cadherin was an independent predictor for progression (HR 3.9, *p*= 0.042) but not for recurrence (57). The gain of N-cadherin expression predicted RFS (HR 1.39, *p*=0.007) in a large retrospective multicenter study that included 827 patients (58). Several years earlier, the same prognostic value of N-Cadherin had been detected (HR 5.88, *p*=0.02) with an independent association between E-cadherin loss and RFS (HR 4.15, *p*=0.02) (59). These two adhesion molecules were also investigated by Liu et al., but they reported that only E-cadherin expression was related to recurrence (60).

In a retrospective study of 233 pTa BC, 27 patients had a progression: 71% of them showed aberrant staining of E-cadherin, in contrast to only 40% without progression (*p*= 0.004). At survival analysis, aberrant E-cadherin expression was a significant predictor of worse PFS (*p* = 0.005). In multivariate analysis, strong E-cadherin staining was independently associated to progression (p = 0.033) (61). Later, a study conducted on 226 pT1 HG BC from TUR confirmed the association of aberrant E-cadherin expression and progression (*p*= 0.045) and showed the same trend at multivariate analysis (HR 0.45, CI 0.19–1.06, *p* = 0.068) (62).

In a recent series of 342 NMIBC, patients with high expression levels of E-cadherin showed a better OS compared to patients with low and intermediate expression (*p*= 0.001), although no association with recurrence was found (32).

As for CIS, a retrospective series of 53 patients demonstrated that E-cadherin loss was independently associated with recurrence (HR 2.8, *p*=0.019), progression (HR 6.6, *p*=0.002) and CSS (HR 4.67, *p*=0.025) (63).

Altogether, evidence supports the hypothesis that E-cadherin expression plays a protective role in NMIBC.

### 3.5 Survivin

Survivin is a member of the apoptosis inhibitors family and plays a regulatory role in controlling cell division (64). Contrarily to non-tumoral tissues, it is overexpressed in human malignancies (65, 66), and has been proposed as a potential marker for early diagnosis of BC, especially for NMIBC (67). In BC treated with RC (any pT stage), early studies demonstrated that survivin expression was a predictor of RFS, PFS, CSS and OS (68, 69),. In a retrospective series of 74 NMIBC, survivin overexpression – using a quantitative evaluation – was associated with recurrence (HR 2.50, 95% CI 1.09-5.69, *p*= 0.02) and progression (HR 3.87, 95% CI 1.13-13.24, *p*= 0.03) on multivariate analyses (70). More recently, Breyer et al. investigated 233 pTa BC, 27 of which had a progression; 92% of them showed overexpression of survivin (based on a qualitative interpretation), compared to 61% in non-progressing patients (*p*= 0.001). Moreover, overexpression of survivin predicted a worse progression-free survival (PFS) (*p* = 0.003) (61). In 2020, multivariate analysis conducted on 134 patients confirmed the association of the intensity of survivin staining – using Allred score – and recurrence (HR 1.451, 95% CI 1.078–1.955, *p*= 0.014) (27).

Evidence suggests that overexpression of survivin predicts a worse outcome in NMIBC patients. Nevertheless, validation studies are still missing, and the definition of overexpression is based on diverse evaluation criteria thus far.

### 3.6 Androgen receptors

The higher incidence of bladder cancer in men and the worse outcome showed by female patients have suggested that this tumor might be endocrine-related (71, 72),. In particular, literature has focused on the role of the androgen receptor (AR) in both carcinogenesis and progression of bladder cancer (73–77). In mice exposed to carcinogens, Hsu et al. reported a lower incidence of bladder cancer and a better survival in subjects with AR ablation in the bladder (74). In men with primary bladder cancer and synchronous prostate cancer, a retrospective analysis showed that those under androgen deprivation therapy for prostate cancer had a longer RFS for bladder carcinoma (77). Different studies described that a change in AR expression levels (high or low expression) seems to be associated with a worse outcome and progression of bladder cancer (78–81). Interestingly, a recent retrospective analysis showed a significantly higher expression of AR mRNA in NMIBC, as compared to MIBC (82).

In 2017, Sikic et al. studied AR mRNA expression in 296 patients with pT1 NMIBC. High AR1 mRNA expression was associated with longer RFS (*p*=0.0007), PFS (*p*=0.0420) and cancer-specific CSS (*p*= 0.0050) at K-M estimates and was an independent predictor for RFS (*p* = 0.0029) and CSS (*p* = 0.0119) at multivariate Cox regression analysis (83). This evidence was recently confirmed by a further retrospective study, in which high AR mRNA expression was also associated with a longer OS in women (*p* = 0.011) and better CSS in males (*p*= 0.044). Interestingly, younger patients showed a longer RFS (*p* = 0.021), CSS (*p* = 0.014) and OS (*p* = 0.007) when high AR expression was detected (84). Another retrospective study conducted on 53 patients with NMIBC demonstrated that low AR mRNA expression was an independent predictor of RFS at multivariate analysis (HR 0.202, 95%CI 0.048–0.841, *p* = 0.028). It also described a tendency to have longer median RFS for the group with high levels of expression (*p* = 0.112) not reaching significance, possibly due to limited number of events and short follow-up (*n* = 10, 9.04 months) (85). As for immunohistochemical AR expression, it was investigated in 40 patients with NMIBC and associated with lower risk of first (HR 0.27, 95%CI 0.084–0.829, *p* = 0.022) and multiple (HR 0.39, 95%CI 0.161–0.927, *p*= 0.033) recurrences in NMIBC at multivariate analysis (86).

Altogether, literature suggests that AR expression in NMIBC has a protective role from recurrence of disease and predicts a better outcome in both men and women. Nevertheless, more evidence and on larger cohorts is needed to better understand this phenomenon, in addition to a comparison between AR mRNA and IHC expression.

### 3.7 Immune and inflammatory biomarkers

Immunotherapy for urothelial carcinoma of the bladder is now routine in clinical practice, as evidence suggests an immunogenic activity of bladder cancer. Therefore, this tumor represents a good model for the investigation of local antitumor responses of the immune system (87).

#### 3.7.1 TILs and other tumor associated leukocytes

The role of tumor-infiltrating lymphocytes (TILs) in NMIBC has been investigated in the landscape of tumor microenvironment. In a retrospective study conducted on low-grade NMIBC not treated with BCG, recurrence was associated with high CD3+ (OR 5.8280; *p*=0.0102) and CD8+ (OR 5.3257; *p*= 0.0092) TILs levels at multivariate analysis (88). Another paper investigated the expression of CD103 – a marker of tissue resident memory CD8+ T cells – in 302 urothelial carcinomas, 212 of which were non-muscle-invasive. Density of intratumoral CD103+ TILs showed a possible role in predicting a more favorable prognosis in terms of RFS (HR 0.6, *p*=0.013) and OS (HR 0.36, *p*=0.002) (87). In contrast, a more recent study of 147 patients with high-grade pT1 bladder cancer failed to find an association with outcome (89).

Recently, tumor-associated macrophages (TAMs) have been studied in a retrospective analysis of 184 patients naïve to systemic chemotherapy/BCG treatment. TAMs were associated to a more aggressive tumor biology. Based on author conclusions, a combination of markers such as CD68, MAC387 and CLEVER-1/Stabilin1 is able to identify most aggressive tumors, and TAMs could become targets for new treatment strategies (90).

Moreover, a recent paper by Dowell et al. stressed the importance of tumor microenvironment in 114 tumor samples collected at the time of diagnosis. They focused on IL-17+ mast cells, finding an increased number of them significantly associated with CIS. The authors supposed that an increased number of IL-17+ mast cells could influence, maybe enhancing, the effect of BCG immunotherapy (91).

Finally, also the function and the role of Natural Killer cells (NK cells) have been investigated in the last decades. NK cells, a relatively rare cell population in the bladder wall, might play an important role during BCG immunotherapy. Using human *in vitro* system and *in vivo* murine tumor model, a BCG-induced cytotoxicity of NK cells was described; at the same time, failure of BCG immunotherapy in mice lacking NK cell activity was observed (92).

Tumor microenvironment is a dynamic phenomenon, and TILs expression can be cyclically promoted or suppressed. Taking this into account, more efforts are needed to reach reliable evidence on this issue.

#### 3.7.2 Cyclooxygenase-2

Cyclooxygenase-2 (COX-2) supports tumorigenesis *via* several mechanisms, including apoptosis inhibition, immune system modulation and angiogenesis promotion, which interfere with endothelial adhesion and cell-cycle molecules (93). COX-2 overexpression has been detected in different tumor types, including BC. However, early studies reported a correlation between COX-2 expression in BC and clinicopathologic and molecular features, but they failed to demonstrate an independent association with clinical outcomes (93, 94),. Similarly, a study conducted on 73 NMIBC (pT1 and CIS) showed no correlation with PFS (*p* = 0.02) and a significant association with RFS only at univariate analysis (*p* < 0.05) (95). Tadin et al. studied 127 patients with low grade NMIBC and discovered a possible protective role for tumor recurrence (HR 0.27, 95%CI 0.14–0.53, *p* < 0.001). The authors hypothesize that, in the initial steps of carcinogenesis2, COX-2 might be associated with the host immune and inflammatory response: a high expression could be a marker of efficient anti-tumor activity. COX-2 expression loss may suggest the need for additional immunotherapy (96).

#### 3.7.3 PD-L1

The programmed death-ligand 1 (PD-L1) is a transmembrane protein expressed by antigen-presenting cells responsible for the deactivation of T lymphocytes (97). Tumor cells might acquire PD-L1 expression and interfere with the surveillance activity of immune cells, promoting tumoral immune escape. PD-L1 based immunotherapy has shown promising results in BC, and PD-L1’s possible additional role as a prognostic biomarker is under investigation. In a study conducted on 334 patients with pT1 bladder cancer, high PD-L1 mRNA expression was correlated with a longer RFS (HR 0.48, 95% CI 0.31-0.72, p=0.0005) (98). However, more recently Aydin et al., analyzing PD-L1 IHC expression, failed to confirm this evidence, although a lower PD-L1 expression was detected in patients with recurrence after treatment with BCG (*p*= 0.012) (99).

**Figure 1:**
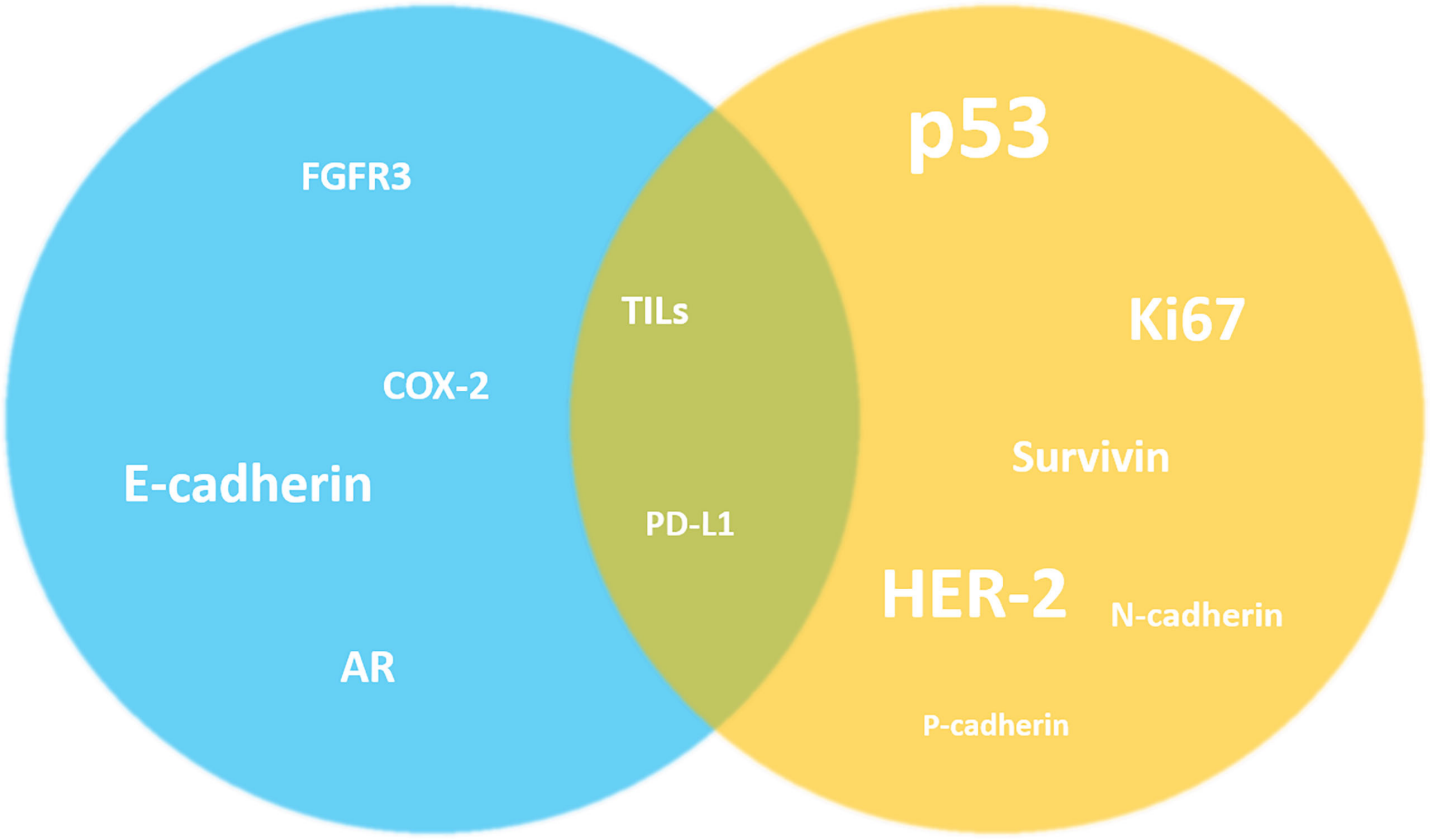
Overall estimation of the possible impact on clinical outcome of the main prognostic biomarkers expression or overexpression in NMIBC. Despite the highlighted conflicting results, it is possible to identify a tendency of the markers to be reported as predictors of better (blue) or worse (yellow) outcome in the literature. For other markers this simplification is not possible (in the middle).The size of the markers graphically reflects the number of studies investigating their prognostic role in NMIBC. AR, androgen receptor; COX-2, Cyclooxygenase-2; FGFR3, fibroblast growth factor receptor 3; HER-2, human epidermal growth factor receptor 2; TILs, tumor-infiltrating lymphocytes.

As mentioned above, the investigation of these potential prognostic biomarkers often provides conflicting results; nevertheless, it is possible to identify for each marker a tendency to be reported as a predictor of better or worse outcome. Overall estimation of the possible impact on clinical outcome of the main prognostic biomarkers expression or overexpression is depicted in **Figure 1**.

### 3.8 Emerging biomarkers for future evaluations

This is a short text to acknowledge the contributions of specific colleagues, institutions, or agencies that aided the efforts of the authors.

In addition to the more investigated biomarkers, other potential prognostic factors for NMIBC have been proposed, all needing further evaluation.

Glutathione (GSH) pathways might be involved in recurrence in NMIBC patients treated with BCG. In a group of 414 NMIBC samples treated with TUR or TUR+BCG therapy, genotyping of 114 single nucleotide polymorphisms in 21 genes of the GSH pathway was performed. This study showed that 7265992 in GSH synthetase was the most significant single nucleotide polymorphisms conferring a worse outcome (p=0.0003) (100).

Robertson et al. attempted to classify NMIBC into different groups based on genomics. Specifically, they studied molecular heterogeneity by RNA sequencing of 73 primary pT1 BC treated with BCG and identified five subtypes; for two of them, they demonstrated worse RFS (101).

The DeltaNp63 isoform of p63 has also been the subject of recent studies for its possible role in NMIBC. Specifically, 134 HG pT1 patients were studied (131 administered with BCG). Using the specific antibody Delta p63, the expression of Delta p63 could be a marker of good prognosis. These patients could benefit from conservative therapies. The strengths of this study were a large cohort and highly heterogeneous therapeutic approaches (102).

In 2017, Sanguedolce et al. assessed HER-2 and microsatellite instability factors MLH1 and MSH2 in 67 pT1 HG BC. Loss of MLH1 immunohistochemical expression was found to predict PFS only in patients treated with BCG; an incrementally worse survival was also correlated with the increase of the number of altered markers. At multivariate analysis, the number of altered molecular markers was the only predictor of both RFS and PFS (*p*=0.0004, *p*=0.0054) (52).

To better understand the role of human leukocyte antigen DR alpha chain (HLA-DRA) in BC tumorigenesis, Piao et al. investigated the expression of HLA-DRA mRNA in tissue samples from both NMIBC (96 patients) and MIBC (43 patients). Interestingly, a higher expression of HLA-DRA was found in HG NMIBC, as compared to low grade NMIBC (*p*<0.05). A high expression of HLA-DRA was also found in NMIBC that progressed to pT2 stage. Moreover, low expression of HLA-DRA in NMIBC was associated to a better PFS (*p*=0.004) (103).

Mutation of the tumor suppressor gene *STAG2* has been frequently detected in BC, especially in NMIBC for which it has been suggested as a predictor of progression (104). More recently, STAG2 immunostaining and combined with other prognostic biomarkers in 297 NMIBC. Low-grade NMIBC with no expression of STAG2 were less likely to progress than positive ones (*p*=0.008) and the same was demonstrated for patients with AUA intermediate and high-risk tumors (*p*=0.02) (105).

In a cohort of 86 pT1 HGBC, Muilwijk et al. found an association between tumor expression of Fibroblast activation protein-α (FAP) – a surrogate marker for cancer-associated fibroblasts – and progression in pT1 BC. Patients who progressed to pT2 showed a higher expression of FAP as compared to non-progressing patients (106).

Assessing possible genetic biomarkers able to predict NMIBC behavior, a recent study included the evaluation of *BUB1* – a mitotic checkpoint serine/threonine kinase – gene expression in 86 NMIBC by real-time qPCR. *BUB1* overexpression was associated with progression (*p*=0.007) and was found to stimulate proliferation of BC cells in *in vitro* analysis. The authors suggest that BUB1 could possibly be used as a prognostic biomarker in NMIBC (107).

We consider IHC evaluation of these tissue biomarkers the most promising and low-cost routine tool for clinical practice. However, we foster further investigations on homogeneous and large study populations and more efforts to reach a consensus on techniques and cut-offs employed.

## 4 Molecular subtypes

Molecular subtypes have been described previously in MIBC, with promising prognostic stratification. Moreover, chemotherapeutic response varies across the molecular subtypes of bladder cancer (108). In fact, basal tumors are associated with a more aggressive behavior in comparison to luminal cancers, while putatively responding better to chemotherapy than luminal ones (109).

However, far less is known about the impact of these molecular subtypes in NMIBC.

Initial data in non-muscle-invasive carcinomas of the upper urinary tract indicate that luminal-like tumors are associated with an unfavorable outcome (110). Different and contrasting results are found in NMIBC: Dadhania et al. compared different molecular cohorts (MDA, Lund), finding a worse CSS and OS for Basal-non-p53 subtypes (111). A molecular profiling study by Mo et al. that considered both MIBC and NMIBC using five gene expression data sets drew similar conclusions, observing higher disease progression for basal subtype in NMIBC (112). Rebola et al. evaluated 147 NMIBC with IHC, using CK5/6 and CK20 markers. They found that high grade pTa/pT1 exhibited a worse CSS, RFS and PFS in luminal subtype. On the contrary, pTa low grade tumors showed a worse CSS when expressing a basal phenotype (113). Finally, the retrospective work of Breyer et al. studied RT-qPCR and IHC in pT1 BC, finding a worse PFS, CSS and RFS for luminal subtype (114). These conflicting results may be explained by the different approaches for defining the different molecular subtypes and may be attributed to the non-homogenous comparison between study populations.

Moreover, Hedegaard et al. evaluated 460 NMIBC, dividing them into three classes after gene expression assays and finding a worse PFS for class 2 tumors (“Infiltrated” or “Genomically Unstable”) (6). To validate a previously developed 12-gene progression score to predict progression to MIBC, Dyrskjøt et al. studied 750 patients affected by NMIBC. In this multicenter prospective study, they observed a worse PFS for “high-risk” molecular subtype (115). Tan et al. studied 2411 NMIBC and MIBC from a meta-cohort, finding a better OS for papillary-like subtype and a worse OS for squamous-cell carcinoma-like in NMIBC (116).

A recent work by Breyer et al. studied 255 pT1 cancers after instillation therapy: a better RFS and PFS for Luminal-A subtype was highlighted, while Luminal-B tumors showed the worst RFS and PFS (50).

**Figure 2:**
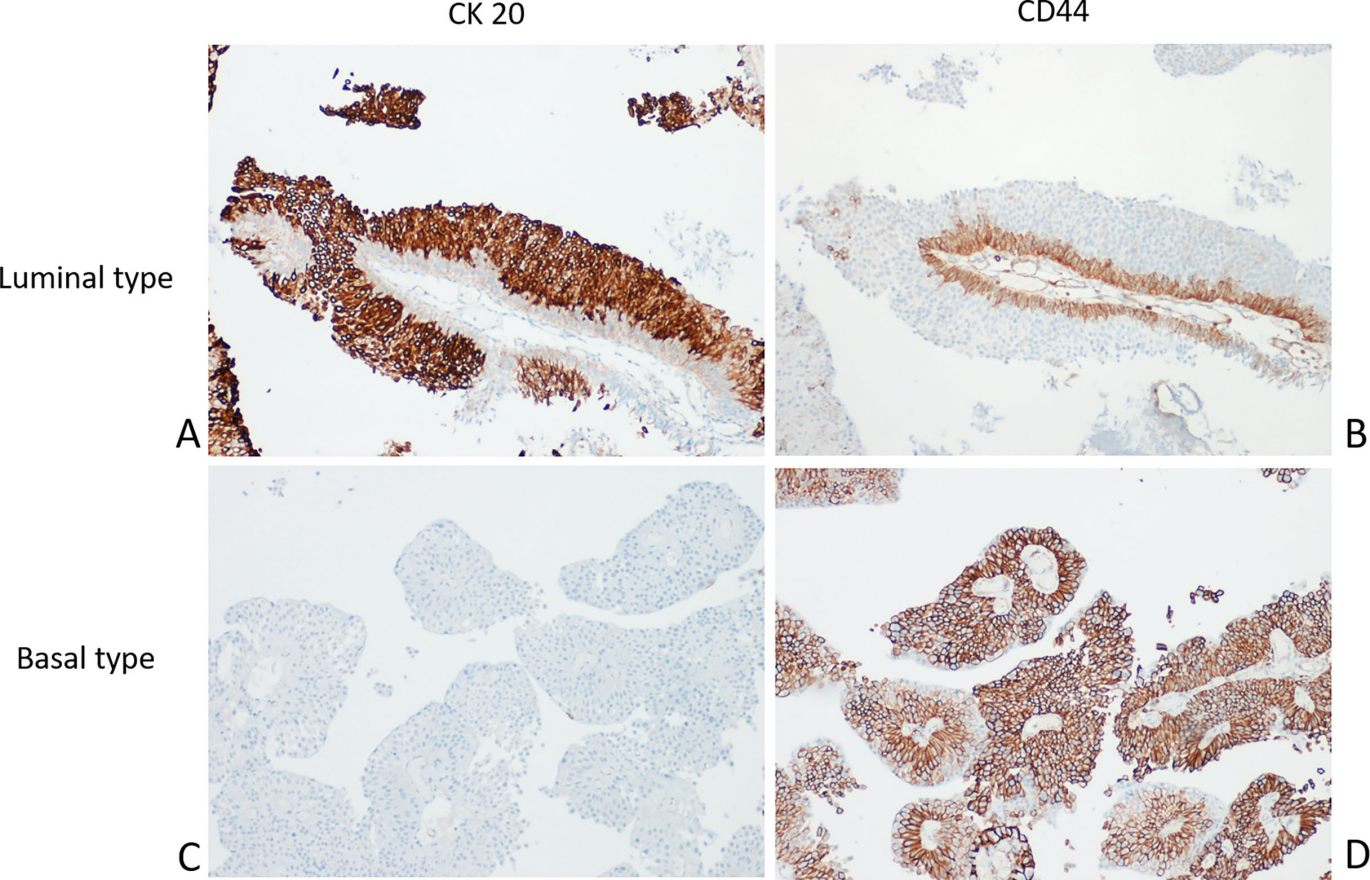
Molecular phenotyping with immunohistochemistry in NMIBC. A prototypical example of Luminal type urothelial carcinoma (UC) with the Luminal marker CK20 **(A)** expressed in almost all tumor cells; in contrast, the Basal marker CD44 is limited to the basal layer **(B)**. Conversely, a typical Basal type UC showing negativity for CK20 **(C)** and diffuse CD44 expression **(D)**.

No reliable data on molecular subtyping are currently available to predict the disease course of CIS (117) or to identify patients who will respond to BCG. For this reason, whole-genome studies have been and continue to be performed for NMIBC with the aim to identify an association with clinical outcomes (118). **Figure 2** shows a paradigmatic example of immunohistochemical stratification in BCs based on most relevant protein expression suggested by recent authors, also adopted in WHO 2016 (5).

## 5 Predictive biomarkers in NMIBC

### 5.1 Biomarkers of BCG response

#### 5.1.1 Immune cell response

##### 5.1.1.1 PD-L1 and PD-1

The standard treatment of patients with high-risk NMIBC is transurethral resection of bladder tumor (TURBT) followed by Bacille Calmette Guerin (BCG) instillations up to three years after biopsies (117). Alternatively, in high-risk tumors, radical cystectomy could be the choice for BCG-unresponsive NMIBC patients.

PD-1 (programmed cell death-1) and PD-L1 (programmed cell death ligand 1) are immune checkpoint proteins that act as co-inhibitory factors that can halt or limit the development of the T cell response. Antibodies targeting these proteins promote antitumor T-cell immunity by blocking inhibitory signals generated by these proteins. In 2016-2017, the Food and Drug Administration (FDA) approved several monoclonal anti-PD-L1/anti-PD-1 antibodies (i.e. atezolizumab, durvalumab, avelumab, nivolumab and pembrolizumab) for patients with locally advanced or metastatic UC that had recurred following platinum-based chemotherapy (119).

Currently, a standard of care protocol for patients who are unfit for surgery or refuse a cystectomy is needed. In 2020, the United States Food and Drug Administration (FDA) approved immune checkpoint inhibitor (ICI) pembrolizumab (anti-PD-1) for the treatment of Bacillus Calmette-Guerin (BCG)-unresponsive, high-risk, non-muscle-invasive bladder cancer (NMIBC) who are ineligible for or have refused cystectomy (120).

At present, no biomarkers able to predict the response to ICI for NMIBC have been validated. However, a preliminary study of 128 patients with Ta/T1 urothelial carcinoma with co-presence of CIS evaluated the efficacy of atezolizumab in a phase II trial and demonstrated an 18-month event free survival rate of 29% (90% CI 22-36%). The 18-month actuarial event free survival rate in 54 patients with Ta/T1 disease was 45% (90% CI 34, 57%) (121).

According to Pierconti et al., among a cohort of 65 cases with primary CIS, PD-L1 expression evaluated through immunohistochemistry (IHC) could identify patients unresponsive to BCG treatment (122).

Moreover, expression of CD4 and CD8 molecules correlates with response to BCG treatment in patients with NMIBC. Specifically, in a pilot study of 40 patients, CD4 levels significantly correlated with longer PFS after BCG treatment. Although promising these results need further investigation due to the limited number of patients involved (123).

In a preclinical study using murine models, CD4+ cells are found to be recruited in the tumor microenvironment but not activated after BCG. One promising approach to improve BCG efficacy in the future may be to activate these recruited T cells (124).

According to another recent paper, positive response to BCG treatment seems to be correlated with low serological levels of CD4 and CD8 and simultaneous high density of the same lymphocytic populations in neoplastic tissue, especially of non-exhausted CD8 subtype. In contrast, failure of BCG treatment correlated with the presence of exhausted CD8. This might suggest that PD1/PD-L1 exhaustion functions as a potential pathway in patients unresponsive to BCG (125).

In a study of association between PD-L1 expression and response to BCG, 63 pretreatment NMIBC cases (31 BCG responders and 32 BCG non-responders) were considered. Baseline PD-L1 expression was observed in 25%-28% of non-responders and 0%-4% of responders (P < 0.01). Moreover, the concentration of CD4+ T cells in pretreated patients was very low among PD-L1+ non-responders and high among PD-L1- non-responders (P < 0.01). High expression for PD-L1 could predict an unfavorable response to BCG and could be used to guide therapeutic decisions. However, this work highlights several limitations, mainly the small size of tumor samples, which did not always allow for adequate setup for TMA (126).

Recently, the possible use of immunomodulatory therapies to reinforce the effects of radiation therapy has been investigated. Emerging evidence by some authors suggests that the generation of antitumor immune response might play an important role in the effectiveness of radiation therapy. This is confirmed by a preclinical murine *in vivo* work followed by subsequent *in vitro* culture analysis, demonstrating that fractionated radiotherapy leads to an up-regulation of PD-L1 in tumor cells due to the production of CD8+ T lymphocytes induced by interferon gamma (INF-g). Hence, blocking the PD-1/PD-L1 axis could increase the immune response in association with fractionated radiotherapy (127). Other authors have reached similar conclusions (128).

##### 5.1.1.2 Cytokines

After its endocytosis through the urothelium, BCG promotes a cascade of events culminating in the activation of interleukins and interferon gamma. The latter attracts neutrophils, macrophages and CD4/CD8 T lymphocytes, which ultimately results in the elimination of tumor cells (129).

A study of 38 patients with CIS tried to identify the factors capable of distinguishing responder and non-responder patients to BCG before treatment. It was found that patients with a polarized Th1 response prior to BCG treatment were non-responders. The authors speculated that BCG or other immune modulators that activate a Th1 response do not enhance a microenvironment already polarized toward Th1. In addition, they evaluated a Th2-type response with histopathological methods, (i.e., Th2-polarized/Th1-polarized lymphocyte ratio, tissue infiltration of eosinophils and eosinophil degranulation levels), developing an immunohistochemical algorithm to predict the response to BCG. According to the authors, a Th2 response correlates with outcome after BCG: only patients with a Th2 response benefit from BCG therapy. The limitations of this study are the small sample size and the retrospective nature of the patients (130).

Pichler et al. reached similar conclusions after evaluating the Th2-polarized tissue response in an immunohistochemical analysis of 40 patients with CIS, pTa and pT1 BC. The authors found a positive correlation between the Th2 response and the BCG outcome, showing that the increase in CD4 lymphocytes is associated with a better RFS. However, post-BCG tumor tissue analysis appears to be necessary to confirm the shift in favor of Th1 in responders. Increased expression of GATA-3, a Th2 response transcription factor, correlates with increased T-Bet lymphocytes in the immune microenvironment of 23 BCG-treated patients; increased lymphocytes presenting these features are associated with prolonged RFS. The strength of this study is the prospective approach and the evaluation of immunohistochemical, serum and urinary expression of samples collected at 10 different time points: before BCG administration, during BCG induction and during follow-up (3,6,9 months) (131).

#### 5.1.2 Molecular analyses, tumor mutation burden

The number of somatic missense mutations present in the baseline tumor sample is called tumor mutational burden (TMB). In recent years, it was found to be a biomarker of tumorigenesis and immune response, as it leads to the formation of neo-antigens that activate T cell immunogenicity to inhibit tumor cells (132, 133).

A recent prospective work evaluated 105 patients with newly diagnosed and not previously treated NMIBC. Targeted exon sequencing analyses (NGS with 341 and 410 cancer-associated gene panels) were performed. The aim was to evaluate the presence of any pre-treatment biomarker capable of identifying which patients will benefit from BCG treatment. It was observed that patients with *ARID1a* mutation have a worse RFS after BCG treatment. According to the authors, this marker could also become a therapeutic target, for example by developing drugs that impact epigenetics (EZH2 methyltransferase inhibitors). In addition, increased mutations in polymorphisms impinging cellular DNA damage repair (DDR) genes, especially *ERCC2*, seems to be associated with a larger mutational burden, which would lead to genomically unstable pathways. The increased mutational burden could also have implications on the effect of systemic therapies with ICI, although further studies are needed to confirm these hypotheses (134). In addition, a recent paper studied 25 primary HR-NMIBC: 10 progressors (T1 HG) and 15 non-progressors (12 T1 HG, 3 Ta HG+CIS). They found that responders to intravesical therapy have a higher rate of mutation compared to progressing tumors (135).

Interestingly, Damrauer et al. performed an RNA-based profiling on 38 NMIBC specimens and found that novel expression signature of an inflamed tumor microenvironment (TME), but not molecular subtyping, could be better associated with improved RFS after BCG immunotherapy. The authors observed that a high immune score was associated with improved RFS after BCG immunotherapy, demonstrating the importance of the pretreatment TME in determining BCG response. On the contrary, the BCG-induced recruitment of immune cells response appears unable to overcome an existing immunologically “cold” TME (136).

### 5.2 Biomarkers of MMC response

A recent paper investigated mitochondria-mediated immunogenic cell death (ICD) as a mechanism of action of MMC. For this purpose, bladder cancer cell lines and NMIBC specimens together with immunization regimens in a syngeneic mouse model were developed. The authors identified a mechanism of mitochondria-induced ICD that may be a critical determinant of chemotherapy efficacy. Based on this study, the tumoral basal abundance of mitochondrial complex I could be considered a potential tool to predict responses to MMC. Indeed, in ICD-resistant tumors for which MMC effects are expected to be limited, the implementation of a more aggressive treatment – intravesical BCG in combination with metabolic modulators – may be preferred (137).

Regarding TMB, a prospective study evaluated the possible association between SNPs contained in *GST1 P1*, *GST01*, *GST02* and *ABCB1* genes and response to therapies in NMIBC. To this aim, a total of 244 patients were enrolled: 130 treated with Epirubicin and 114 with Mytomycin. The authors found that the expression of *GSTP1* and *GST01* was associated with recurrence in patients treated with intravesical Epirubicin but not with those treated with Mytomycin C. Moreover, these polymorphisms seem to be associated with Epirubicin-induced toxicity. According to the authors, selected SNP genotypes could become the basis for individualized future therapies (138).

### 5.3 FGFR, RRM1 and EZRIN

In recent years, new trials have been developed and initiated to test the effect of tyrosine kinase inhibitor drugs, as a proportion of NMIBC are shown to have somatic mutations in these biochemical pathways, the most important being FGFR3. The FGFR1-4 inhibitor Erdafitinib is already FDA-approved for the treatment of patients with metastatic urothelial carcinoma, and preliminary efficacy and safety are now being evaluated in a phase II study enrolling NMIBC patients with somatic *FGFR3* mutations or fusions (NCT04172675) (139). In addition to FGFR inhibitors, other tyrosine kinase inhibitors have been studied. Dovitinib is a multi-kinase inhibitor that has been recently studied in a phase II study (N = 13) enrolling BCG-unresponsive NMIBC patients with FGFR3 somatic mutations. Only patients with both *FGFR3* mutations and increased FGFR3 expression showed CR at six months (33%), whereas patients with only FGFR3 expression demonstrated no tumor response (140). Gemcitabine is a deoxycytidine analog. Active forms of gemcitabine inhibit DNA synthesis by incorporating into the DNA chain or by inhibiting RRM1 activity. Preclinical and clinical data indicate that high levels of RRM1 protein are associated with gemcitabine resistance in several cancers. A retrospective study enrolled 162 patients with histologically confirmed NMIBC and intermediate/high risk disease. All patients received intravesical gemcitabine monotherapy immediately after TURBT. This study demonstrated that high RRM1 expression was observed in less than 30% of tumors and was an unfavorable prognostic factor for PFS. The study has some limitations, including being a single-center study and the relatively small number of patients analyzed for RRM1 (N = 17) (141). In conclusion, new alternative treatment options are emerging for high-risk BCG-unresponsive and BCG-naïve NMIBC patients who are unfit or refuse radical cystectomy.

In 2009, Ezrin, a protein involved in cell adhesion, migration mechanisms and epithelial-to-mesenchymal transition, was found to have a prognostic value (142). One study evaluated a total of 294 patients: of these, 152 were treated with BCG, 63 with MMC and 79 with a combination of Epirubicin and Interferon alpha2b. By multivariate analysis, Ezrin was the only factor that correlated with BCG treatment failure (p=0.022) (143).

**Figure 3:**
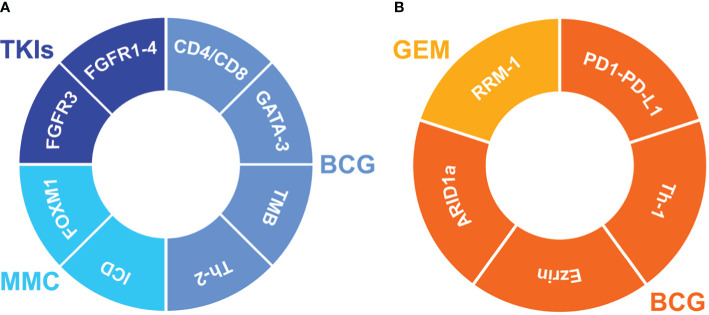
Impact of the predictive biomarkers on the clinical outcome in NMIBC. Markers improving the tumor outcome **(A)** during treatment with BCG (bacillus Calmette-Guérin), MMC (mitomycin C) or TKIs (tyrosine kinase inhibitors). Markers worsening the tumor outcome **(B)** during treatment with BCG or GEM (Gemcitabine). FGFR3, fibroblast growth factor receptor 3; FGFR1-4, fibroblast epidermal growth factor receptor 1-4; CD4/CD8, CD4/CD8 T-Lymphocytes; GATA-3, GATA binding protein 3; TMB, Tumor Mutational Burden; Th-2, T helper-2 lymphocytes; ICD, mitochondria-mediated immunogenic cell death; FOXM1, Forkhead Box M1; RRM-1, ribonucleotide reductase subunit M1; PD1-PD-L1, Programmed Cell Death Protein 1/Programmed death-ligand 1; Th-1, T helper-1 lymphocytes; ARID1a, AT-rich interactive domain-containing protein 1A.

A summary of most relevant tissue predictive markers is depicted in **Figure 3**.

## 6 Discussion

The employment of standardized and reproducible biomarkers to stratify patients and drive therapeutical decision-making is still an unmet need. This is mostly due to intra- and inter-tumoral heterogeneity, which occurs at multiple levels and is best demonstrated in tumors with variant histology. Nevertheless, ancillary methods beyond histology could facilitate clinical evaluation and better identify patients who will recur or progress from NMI to MIBC, eventually selecting patients who might need early radical intervention. Thus, the role of genetic and molecular properties is a growing body of research.

In this review, we outlined and summarized the most relevant published literature on tissue biomarkers in NMIBC with prognostic and predictive purposes. Although promising, most of these studies presented some limitations. Cohorts analyzed were mostly from mono-institutional populations with a limited number of cases, lacking external validation. Only few studies had a prospective approach, and some used different assessment methods or variable cut-offs, affecting reproducibility. We foster further investigations to validate the role of tissue biomarkers in predicting NMIBC recurrence or progression in daily practice, with a particular focus on cost-effective tools such as immunohistochemistry.

## Author contributions

Conceptualization, writing-original draft preparation, review: PC, CC, and MV; analysis and validation: PC, CC, MV, and MC; editing: DC; supervision: PC. All authors contributed to the article and approved the submitted version.

## Conflict of interest

The authors declare that the research was conducted in the absence of any commercial or financial relationships that could be construed as a potential conflict of interest.

## Publisher’s note

All claims expressed in this article are solely those of the authors and do not necessarily represent those of their affiliated organizations, or those of the publisher, the editors and the reviewers. Any product that may be evaluated in this article, or claim that may be made by its manufacturer, is not guaranteed or endorsed by the publisher.
